# Potential Lipid Signatures for Diagnosis and Prognosis of Sepsis and Systemic Inflammatory Response Syndrome

**DOI:** 10.3390/metabo10090359

**Published:** 2020-09-01

**Authors:** Giovana Colozza Mecatti, Salvador Sánchez-Vinces, Anna Maria A. P. Fernandes, Marcia C. F. Messias, Gabrielle K. D. de Santis, Andreia M. Porcari, Fernando A. L. Marson, Patrícia de Oliveira Carvalho

**Affiliations:** 1 Laboratory of Multidisciplinary Research, São Francisco University, Bragança Paulista, São Paulo 12916-900, Brazil; giovana.mecatti@usf.edu.br (G.C.M.); salvador.vinces@mail.usf.edu.br (S.S.-V.); anna.fernandes@mail.usf.edu.br (A.M.A.P.F.); marcia.cfmessias@gmail.com (M.C.F.M.); gabi.kris.98@gmail.com (G.K.D.d.S.); andreia.porcari@usf.edu.br (A.M.P.); 2 Laboratory of Human and Medical Genetics, São Francisco University, Bragança Paulista, São Paulo 12916-900, Brazil; fernando.marson@usf.edu.br

**Keywords:** sepsis, SIRS, lipidomics, multivariate analysis

## Abstract

Systemic inflammatory response syndrome (SIRS) and sepsis are two conditions which are difficult to differentiate clinically and which are strongly impacted for prompt intervention. This study identified potential lipid signatures that are able to differentiate SIRS from sepsis and to predict prognosis. Forty-two patients, including 21 patients with sepsis and 21 patients with SIRS, were involved in the study. Liquid chromatography coupled to mass spectrometry and multivariate statistical methods were used to determine lipids present in patient plasma. The obtained lipid signatures revealed 355 features for the negative ion mode and 297 for the positive ion mode, which were relevant for differential diagnosis of sepsis and SIRS. These lipids were also tested as prognosis predictors. Lastly, L-octanoylcarnitine was found to be the most promising lipid signature for both the diagnosis and prognosis of critically ill patients, with accuracies of 75% for both purposes. In short, we presented the determination of lipid signatures as a potential tool for differential diagnosis of sepsis and SIRS and prognosis of these patients.

## 1. Introduction

The definition of sepsis, as introduced in 2016, updated several concepts and brought some new ones. Now, sepsis is defined as a life-threatening organ dysfunction caused by a dysregulated host response to infection that includes immune as well as nonimmune responses [1]. All over the world, nearly 6 million people die of sepsis annually [2]. Systemic inflammatory response syndrome (SIRS) is a condition in which the patient presents two of the following signs: tachycardia, fever or hypothermia, leukocytosis or leukopenia and tachypnea. It may also occur in response to various forms of aggression such as infection, trauma or surgery. Almost all septic patients have SIRS, but not all SIRS patients are septic. As an exception to this theory, it has been suggested that there are subgroups of hospitalized elderly patients who do not meet criteria for SIRS on presentation but progress to severe infection and multiple organ dysfunction and death. For this reason, SIRS could be an element of confusion for the diagnosis, management plan or evolution assessment and, eventually, patient prognosis prediction [3].

An insufficient ability to predict sepsis prognosis continues to be an important issue, despite the increasing use of clinical tools [4], severity scores (e.g., the Acute Physiology and Chronic Health Evaluation II (APACHE II) [5], the Simplified Acute Physiology Score III (SAPS III) [6], Sequential Organ Failure Assessment (SOFA), qSOFA (quick SOFA)) and biomarkers (e.g., procalcitonin (PCT), presepsin and C-reactive protein (CRP) [7]). Since sepsis has a clinical diagnosis of great biological complexity, not much progress has been made towards an effective predictive approach to sepsis in terms of specific diagnosis or prognosis [8]. The characterization of molecular mechanisms of events associated with sepsis, such as organ failure, treatment response evolution and death, are also not well understood [9].

SIRS suffers from a lack of precision in defining the factors that produce a disease (infectious or not), its evolution and the patient’s outcome [1]. Satisfying the two minimum criteria for a SIRS diagnosis is relatively common in some infectious or non-infectious diseases (i.e., pancreatitis or trauma) [10]. However, the importance of assessing the presence of these factors has generated different results and, at the very least, they are intriguing due to the evident selectivity in terms of when and where they are most useful [11].

Recent omics techniques have facilitated high-throughput profiling of pathology-related signatures and biomarkers in biological fluids [12]. Lipidomics is one of the most recent, rapidly developing and promising approaches [13]. Lipidomics studies the state of the lipid molecular phenotype, reflecting the functional “landscape” of lipid activity in cells and tissues [14]. For this reason, clinical lipidomics offers the possibility of elucidating the strategic roles of lipids in disease and the immune system [15], identifying biomarkers and developing new therapeutics [16]. Recent studies have shown the potential diagnostic [17] and prognostic [18] roles of lipidomics in sepsis patients. Some promising results from the existing literature have evaluated differences between control and sepsis patients [17,19] and between patients who survived sepsis and those who did not [20]. A less frequent comparison covers the difference between sepsis and SIRS by evaluating the potential for the diagnosis or prognosis of either [20,21]. The terms outcome and prognosis have been used here as if they were synonyms and are understood to be the final survival report of each patient [22].

In this prospective study, the lipid profiles obtained from plasma samples of patients diagnosed with sepsis were compared with patients diagnosed with non-septic SIRS in order to identify sepsis-specific biomarkers. These lipids with potential for differential diagnosis were assessed as prognostic biomarkers and their putative biological roles were summarized.

## 2. Results

### 2.1. Subject and Clinical Data

Table 1 shows a statistical comparison of the two groups for the baseline characteristics of the participants involved in this study. No significant differences were found between the demographic characteristics of the groups. Other prognostic scores did not present a statistically significant difference. Almost no comorbidities were present in the SIRS group; this can be explained by the epidemiological characteristics of this group of patients as they were almost all victims of poly-trauma. A higher frequency of comorbidities is expected in patients in the sepsis group [23], leading to a statistically significant difference for systemic hypertension and for diabetes mellitus. The other comorbidities were less frequent in our patients with sepsis, so there were no statistically significant differences, even in the absence of the comorbidities in the SIRS group. No significant differences were found in the frequency of organ dysfunctions between the groups, since both diagnoses can lead to the occurrence of these dysfunctions. None of these variables had a statistically significant effect on the multiple linear regression model for diagnostic classification (Supplementary Table S3). All of the non-survivor patients in the study died during their intensive care unit (ICU) stay.

### 2.2. Analysis of Plasma Samples

**Figure 1:**
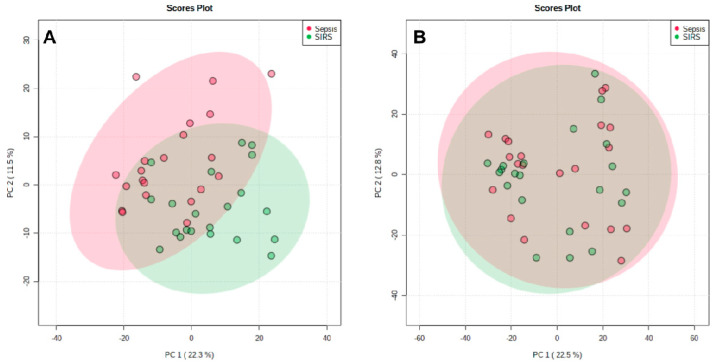
Principal component analysis (PCA) score plot between the first 2 principal components (PC) for negative ionization mode (**A**) and positive ionization mode (**B**). Areas of 95% confidence are highlighted in red and green. Variance explanation (**%**) for each PC is indicated.

In this study, 42 samples were assessed: 21 plasma samples from male patients diagnosed with sepsis and 21 plasma samples from male patients diagnosed with SIRS. After applying quality control (QC) and non-QC filters and making corrections, final numbers of 733 features for negative ion mode and 1703 features for positive ion mode were obtained. The obtained lipidome data were assessed using principal component analysis (PCA) for both negative (Figure 1A) and positive ionization modes (Figure 1B). In negative mode, both groups presented very close individual profiles which impeded complete separation of groups. In positive mode, there is a total overlap of the groups. Supplementary Figure S1 shows PCA for samples and QC, where high-quality data depict QC samples in clusters tighter than those observed for biological samples [24].

**Figure 2:**
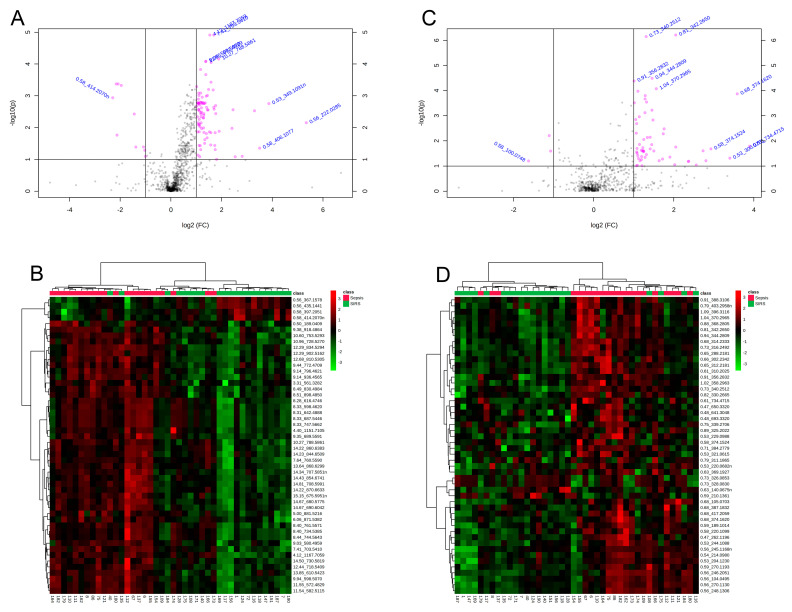
Volcano plot of features for negative ionization mode (**A**) and positive ionization mode (**C**). Heat map of clustered differential features and samples for negative mode (**B**) and positive mode (**D**). In the volcano plot, highlighted features with adjusted *p*-value of 0.05 and log (fold change) of 1. Heatmap depicts top 50 features with lowest adjusted *p*-values.

Other descriptive analyses, such as volcano plot and heatmap of clustered intensities, were performed for all the features. The results are represented in Figure 2 for both negative and positive modes. These descriptive results show that, despite the difficulty in differentiating groups by PCA, it is still possible to determine features with differential abundances.

#### 2.2.1. Analysis of Lipid Signatures for Diagnosis

In order to identify the most relevant features in the task of correctly classifying the samples by diagnosis (sepsis or SIRS), a selection of the lipid signatures was made with prediction models using the random forest (RF) method implemented in MetaboanalystR. The final model for the negative mode (accuracy = 84.7%, area under the curve (AUC) = 0.935) selected 355 features as relevant. The final model for the positive mode (accuracy = 75.7%, AUC = 0.868) selected 297 features as relevant. Receiver Operating Characteristic (ROC) curves for these models are provided as Supplementary Figures S2 and S3.

Matching the obtained list of features from the RF model for negative and positive mode with Lipidmaps and Human Metabolome DataBase (HMDB) databases resulted in the annotation of 33 significant features as possible biomarkers for discriminant diagnosis between sepsis and SIRS (Table 2). Annotated lipids such as L-palmitoylcarnitine, gamma-linolenyl carnitine, linoleyl carnitine and the omega 6 polyunsaturated fatty acid arachidonic acid were found in higher abundance in the sepsis patient’s plasma and were significant contributors to differentiation among sepsis and SIRS. The predictive importance of these putatively identified lipids was evaluated in subsequent analyses.

**Figure 3:**
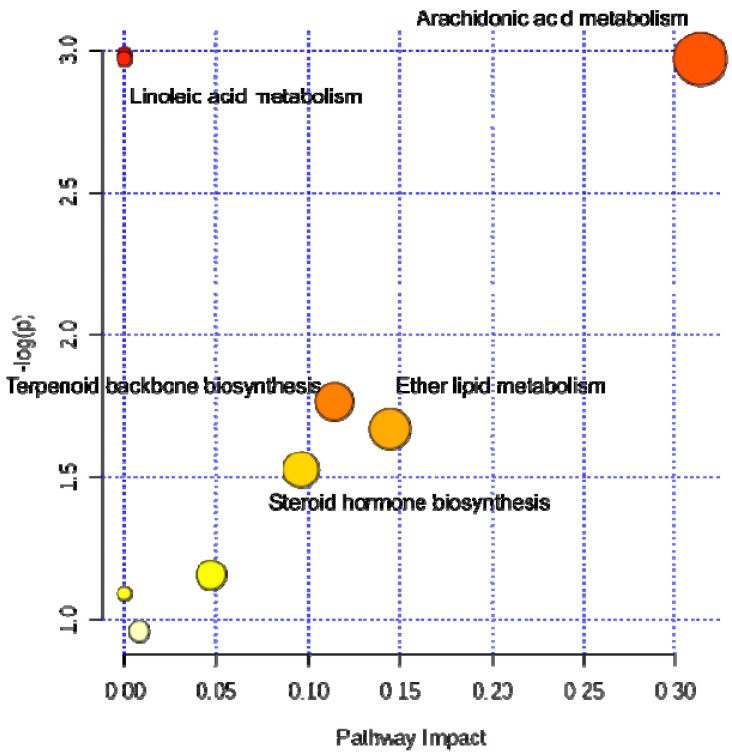
Summary of pathway analysis.

Figure 3 shows the metabolic pathways most associated with the lipids found to be relevant. A large impact on the pathway is related to the importance of the compound within the metabolic network evaluated; a higher log (p) (or lower *p*-value) indicates the over-representation of the evaluated pathway in relation to the list of compounds consulted. Only 22 compounds were found in the HMDB database. Supplementary Table S2 shows information on the matched lipids and statistics of the enriched pathways.

#### 2.2.2. Performance Evaluation of Diagnostic Lipid Signatures Used for Prognostic Prediction

**Figure 4:**
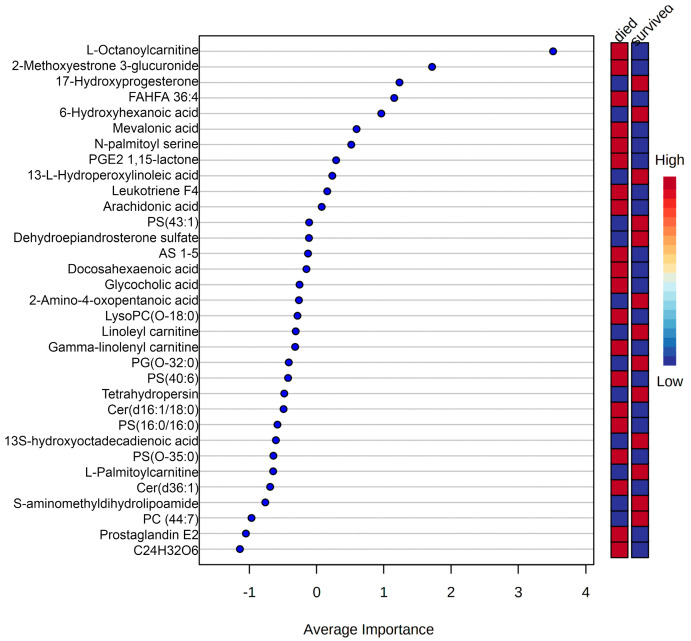
Prognostic classification using identified lipids from diagnosis classification model. Lipids ranked by importance for the random forest based model.

With a more reduced but significant list of features, random forest for multivariate classification was used to assess features′ performances as possible signatures for prognostic classification (Figure 4). This model had an average accuracy of 61.3% and an AUC = 0.676 (see Supplementary Figure S4). Supplementary Table S1 provides a complete list of ranked scores. Although this model presents low accuracy due to the small number of features selected for prognostic classification, its results enabled the identification of the most relevant features for further analysis.

#### 2.2.3. Performance Evaluation of L-Octanoylcarnitine as Diagnostic and Prognostic Predictor

**Figure 5:**
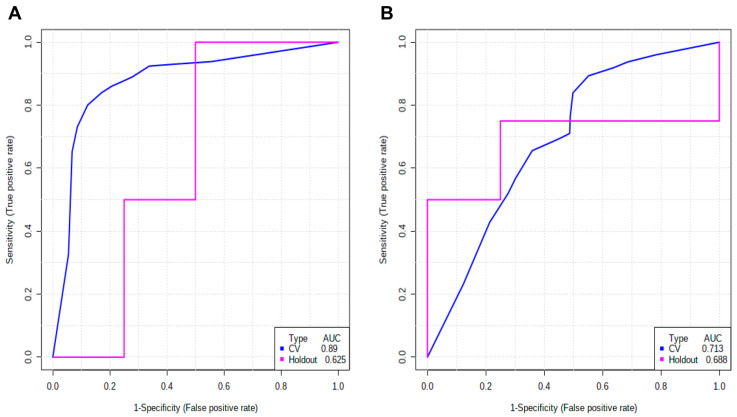
Classification performance of L-octanoylcarnitine for diagnosis (**A**) and prognosis (**B**). In (**A**), the receiver operating characteristic (ROC) curve shows AUC = 0.89 for training/test and ROC AUC = 0.625 for validation (holdout). (**B**) For prognostic classification, it shows the receiver operating characteristic (ROC) curve AUC = 0.713 for training/test and ROC AUC = 0.688 for validation (holdout).

The lipid L-Octanoylcarnitine was found to be the most relevant for the prognostic classification (samples from patients who survived and died), with a notable difference in importance in relation to the other lipids. To evaluate its individual importance in diagnostic and prognostic classification, classification prediction models were used based on random forest. To build the classification models, eight samples were randomly selected and unlabeled (four from each group for each classification) to define a validation group. As a diagnostic signature (Figure 5A), L-octanoylcarnitine obtained an AUC = 0.89, an average accuracy based on 100 cross-validations of 0.848 and accuracy for validation data prediction of 0.75 (6/8), one mismatch per class. As a prognostic signature (Figure 5B), L-octanoylcarnitine obtained an AUC = 0.713, an average accuracy based on 100 cross-validations of 0.658 and accuracy for validation data prediction was 0.75 (6/8), one mismatch per class.

**Figure 6:**
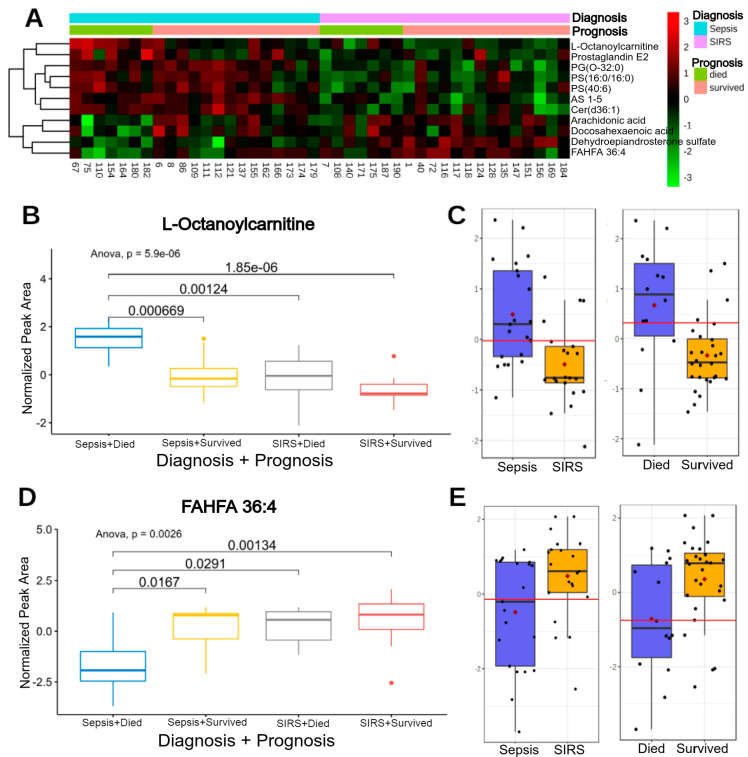
(**A**) Heatmap of standardized intensities of significant lipids obtained by two-way ANOVA. The upper bars indicate the group to which the samples belong (columns). Lipid clusters on the left side. (**B**,**D**) Boxplot of the 2 most relevant lipids. Boxplot of subgroups (diagnosis + prognosis) with statistical significance values obtained by ANOVA and Tukey′s test. Boxplots for diagnosis and prognosis classification for (**C**) L-octanoylcarnintie and (**E**) FAHFA 36:4.

The two-way ANOVA analysis identified seven significant variables for classification by diagnosis (PS (40:6), PS (16:0/16:0), Cer (d36:1), PG (O-32:0), prostaglandin E2, AS 1-5, dehydroepiandrosterone sulfate), two for classification by prognosis (arachidonic acid, docosahexaenoic acid) and two relevant for both classifications: L-octanoylcarnitine and FAHFA 36:4. Figure 6A shows a heatmap of clustered intensities of these lipids for samples grouped by diagnosis and subgrouped by prognosis. Figure 6B plots the differences in intensity by each group for the lipids found to be important for both categories.

## 3. Discussion

Sepsis, one of the major causes of death in the world, is a serious medical condition associated with high incidence and mortality rates [25]. The discovery of differentiators of patients with a high chance of poor outcome should optimize the selection of better treatment strategies. Similarly, early discrimination between sepsis and some other similar clinical condition, such as SIRS, would make better decision making possible by preventing the progression of the disease, even before organ dysfunction. Here, we have shown that the differences in the lipidomes of patients diagnosed with sepsis or SIRS are relevant for the patients’ prognoses. Although differences in the prognosis of patients with sepsis or SIRS, detected either by different omic or clinical approaches [1,17,21,26], have been previously reported; in the present study, we report the co-occurrence of variations in lipid abundance with diagnostic and prognostic potential.

Some species of glycosphingolipids (AS 1–5), glycerophospholipids (PS (16:0/16:0), PS (40:6), PG (O-32:0), PG (O-32:0)), N-acylsphingosines (Cer (d16:1/18:0), Cer (d36:1)), prostaglandins (prostaglandin E2), lineolic acids (13S-hydroxyoctadecadienoic acid), phosphatidylcholine (PC (44:7)) and acylcarnitines (L-octanoylcarnitine, L-palmitoylcarnitine) were more abundant in the sepsis diagnosed group when compared with the SIRS group, while some species of sulfated steroid (dehydroepiandrosterone sulfate,), fatty acid esters of hydroxy fatty acids (FAHFA 36:4), were more abundant in cases with SIRS when compared with the sepsis cases. When comparing the groups of survivor and non-survivor patients, no univariate adjusted statistical difference (false discovery rate (FDR) *p* < 0.05) was found, but multivariate relevance was found, especially in L-octanoylcarnitine (more abundant in non-survivor patients) and FAHFA 36:4 (more abundant in survivor patients). In addition, the last two compounds present interaction between the two groups, with abundances codependent on prognosis and diagnosis.

Glycosphingolipids (GSLs) are a subclass of sphingolipids with glycans exposed to the extracellular space. These lipids are abundant components of the cell membrane [27]. GSLs are related to many biological processes including infections by specific pathogens as binding receptors at the surface of host cells [28]. GSLs play a role in immune cell function as a signal transducer (i.e., toxins or IgM antibodies) or in binding lipid rafts to trigger chemotaxis, phagocytosis and phagolysosome formation [28] and are involved in regulatory aspects of T cell biology [29]. Some clinical uses for GSLs, such as lipid-rafts for signaling the presence of pathogens, and pharmacological reduction of GSL are being actively studied [30]. However, so far, there have been no studies that describe or associate AS 1-5, a glycosylated N-acylsphingosine, with immune response, inflammation or infection so far.

Glycerophospholipids or phosphoglycerides are lipids with hydrophobic regions composed of two fatty acids linked to glycerol. Sphingolipids are lipids with a single fatty acid linked to a fatty amine, sphingosine. Both lipids are the main components of biological membranes. A wide variety of these compounds have been reported as differentials in assessing septic mortality [21] or in differentiating stages of sepsis and SIRS [20]. These compounds present an increase in abundance related to the severity of sepsis, being more abundant in septic shock and non-differential in non-infectious SIRS [31]. A confounding factor when analyzing these compounds is the variability of their abundance, sometimes decreased in sepsis, depending on the type and focus of infection (i.e., decrease in lysophosphatidylcholines in community-acquired pneumonia) [32]. This high variability has made its biological interpretation difficult. Interestingly, the compounds of this class identified in our study have a higher mean abundance in sepsis, although with weak univariate statistics but relevance in multivariate differentiation. These compounds are largely associated with lipid peroxidation, whose products may have pro-inflammatory and protective activity against infection [33].

Ceramides play essential roles in cell signaling and contrasting roles within cellular metabolism. Ceramide is involved in cellular responses related to stress, autophagy and apoptosis, whereas S1P, another bioactive lipid of the sphingolipid pathway, stimulates cell survival, proliferation and tissue regeneration [34]. However, it is necessary for further investigation to understand the effect of different lengths of acyl chains on this lipid class. Again, sphingolipids participate in the regulation of the phagosome/lysosome fusion, apoptosis or the inflammatory response [35], facilitating bacterial destruction.

Higher average importance for multivariate model and univariate significance of L-octanoylcarnitine and L-palmitoylcarnitine were found in the sepsis group and just low average importance for gamma-linolenyl carnitine and linoleyl carnitine for the same model. The quaternary ammonium compound carnitine and its acyl esters (acylcarnitines) are essential for the oxidative catabolism of fatty acids and thence for maintaining energy homeostasis in the human body. Downregulation of fatty acid oxidation is evidenced by an increased presence of acylcarnitines in plasma [36]. Their accumulation in the plasma is marked in sepsis non-survivors, indicating a possible mitochondrial dysfunction in energy production. Moreover, it was reported that non-survivor septic patients have mitochondrial dysfunction leading to deficient aerobic catabolism [37] and consequently elevated plasma concentrations of TCA cycle metabolites. Unused acylcarnitines are reversely transported to the cytoplasm and then into the plasma [38]. Levels of these lipids were found to be lower in SIRS and survivor patients, as reported by other studies [20]. In the present study, we looked for a particular abundance profile for prognostic and diagnostic classifications: L-octanoylcarnitine presented the highest abundance in non-survivor sepsis patients when compared to survivor SIRS patients (lowest abundance), non-survivor SIRS and survivor sepsis patients. Its importance was evaluated by univariate and multivariate prediction methods (Figure 6), with good predictive performance for both diagnoses and prognoses (Figure 5, which identifies it as a possible lipid signature). This compound is the physiologically active form of octanoylcarnitine, an intermediate fatty acid b-oxidation byproduct. In addition to indicating increased lipid oxidation, L-octanoylcarnitine may indicate increased lipid input [39]. A recent study identified low levels of L-octanoylcarnitine as a biomarker of breast cancer (100% positive predictive value) against samples from healthy individuals, in addition to presenting different levels depending on the size of the tumor, as well as high abundance in tumors with high expression of estrogen and progesterone receptors [40]. This may be related to the high metabolic demand of the tumor. Another study on prostate cancer showed a positive relationship between L-octanoylcarnitine levels and the risk of cancer progression in primary and metastatic samples [41]. There is currently no information that relates this acylcarnitine to sepsis, SIRS or the prognosis of these cases. However, a larger, stratified study covering a wider range of compounds (metabolites and proteins) is needed to infer the biological basis of their variable abundance in the cases presented here.

FAHFA 36:4 is a compound that presented a different pattern to those mentioned above. This fatty acid ester of hydroxy fatty acid was found to be more abundant in samples of surviving patients with SIRS when compared to non-survivors with sepsis (less abundant), survivors with sepsis and non-survivors with SIRS. These lipids are endogenous products present in food and mammalian tissues. To date, more than 16 FAHFA families have been determined. Structurally, each family has different fatty acid and hydroxy fatty acid compositions and multiple isomers by the ester bond position. These compounds have anti-inflammatory and anti-diabetic effects [42]. Although it is not known how they perform their biological activity, recent studies link FAHFA to erythroid nuclear factor 2-related factor 2 (Nrf2) [43]. Their presence is related to resolution or regulation of inflammation, including providing protection against potential infection [44]. Therefore, it is not clear whether the low abundance in patients with sepsis and in non-survivors is a depletion or a result of some altered pathway. No studies have been published that relate FAHFA to the progression and outcome of patients with sepsis or SIRS.

In conclusion, this lipidomics study carried out on plasma taken from male patients with sepsis or SIRS assessed relevant lipids for diagnosing. Then, identified lipids from the previous step were assessed as prognostic signatures. Finally, one relevant lipid, L-octanoylcarnitine, was found to be a promising signature for diagnosis and prognosis. Quantification studies of all relevant metabolites highlighted by this study and their physiological and altered levels in human plasma seem to be an interesting matter for further investigation.

## 4. Materials and Methods

### 4.1. Study Groups

The study samples came from the Universidade São Francisco (USF) Hospital, Bragança Paulista, São Paulo, Brazil. Male patients admitted to the ICU were evaluated. The project was approved by the Research Ethics Committee of the Universidade São Francisco (CAAE:51356315.5.0000.5514) and was developed at the Intensive Care Unit of Universidade São Francisco Hospital. The following inclusion criteria were adopted for the group of critically ill patients: individuals from 15 to 90 years of age admitted to the intensive care unit, either clinical or surgical, in the period. Female patients and patients receiving special diets were not included in the study to avoid gender-related and diet-related lipidomic profile bias [45]. Following SIRS definition criteria [46], 21 male patients with 2 or more signs of SIRS and no suspected or confirmed infection were selected for inclusion in the SIRS group. Patients with organ dysfunction and confirmed infection were selected for inclusion in the sepsis group. Clinical data were collected, including severity score (SAPS III and SOFA on the first day of hospitalization). Clinical and demographic data are provided in Table 1. Additionally, a logistic multiple linear regression model was implemented to evaluate the influence of non-lipidomic variables on the classification (diagnostic) variable.

### 4.2. Sample Collection, Preparation and LC-MS/MS Analysis

Blood samples were collected for daily laboratory monitoring of critically ill patients and aliquots of this material from the first 36 h of hospitalization were used to carry out the analyses of the present study. Labeled ethylenediamine tetraacetic acid (EDTA) blood samples were sent to the Multidisciplinary Research Laboratory of the USF, where the lipidomic analyses were performed. Centrifuged plasma samples were frozen at −80 °C. A mixture of samples from both groups was used as quality control (QC). This pooled sample was divided and extracted along with the remaining samples. CHCl_3_:MeOH solution (2:1, v/v) was used for extraction with 150 mL of plasma sample. Extracted samples were then vortexed for 30 s and centrifuged at 12,000× RPM for 5 min at 4 °C. The bottom organic layer (450 mL) was collected. Nitrogen-dried samples were stored at −20 °C to await analysis. A solution of isopropanol (IPA)/acetonitrile (ACN)/water (2:1:1, v/v/v) was used to reconstitute samples before analysis.

#### 4.2.1. LC-MS Analysis

Following a method previously published by our group [47], untargeted LC-MS analysis was performed using an ACQUITY UPLC coupled to a XEVO-G2XS QTOF mass spectrometer (Waters, Manchester, UK). Liquid chromatography was performed using an Acquity UPLC CSHC18 column (2.1 × 100 mm, 1.7 mm, Waters). The volume of injection was 1 mL. MS^E^ mode was used to separately record positive and negative ion modes in the range of 50–2000 *m/z*. The injection order was randomly defined and QC samples were analyzed after every ten injections.

#### 4.2.2. Data Acquisition and Preprocessing

The peak alignment, deconvolution, selection of possible adducts and compound annotation based on MS^E^ experiments were obtained using Progenesis QI 2.0 software (Nonlinear Dynamics, Newcastle, UK). Search parameters for putative annotation were precursor mass error 5 ppm and fragment tolerance 10 ppm. At this stage, putative identification using LIPID MAPS [48] database and the Human Metabolome Database (HMDB) [49] was defined by fragmentation score, mass accuracy and isotope similarity. Annotation of compounds was classified in accordance with the Metabolomics Standards Initiative (MSI) [50], where ions with some level of match with MS/MS database reached level 2 while compounds putatively identified by exact mass, using the mummichog algorithm, reached level 3. Progenesis QI generated a table of ion intensity by sample and ions. Ions were labeled according to their retention time and mass-to-charge (*m/z*) ratio. Preprocessed data are available as Supplementary Materials files: Spreadsheet_1 Sepsis SIRS negative mode, for negative mode; Spreadsheet_2 Sepsis SIRS positive mode for positive mode.

### 4.3. Statistical Analysis

MetaboAnalystR 3.0 [51], statTarget2 [52] and Bioconductor package manager using R programming language [53], were used to perform statistical analyses. Quality control based signal correction was performed using random forest implementation (QC-RFSC) [54]. According to the “80% rule” [55], peaks present in more than 80% of the samples of each group were kept for further analysis. The K-nearest neighbor algorithm was used to impute the remaining missing values. Further data filtering removed variables with low variance based on the interquartile range (IQR) [56]. Then, the corrected data were log-transformed and normalized using the Pareto scale [57].

#### 4.3.1. Exploratory Analysis

For univariate descriptive analyses, a volcano plot was used to represent features with FDR-adjusted *p*-values < 0.05 using t-test and 2-fold intensity between groups for each *m/z*. Principal component analysis (PCA) was used to distinguish sample cluster distribution in the first two principal components. A heatmap and unsupervised hierarchical clustering of 50 features with the lowest adjusted *p*-value < 0.05 depicts differential peaks.

#### 4.3.2. Analysis of Biomarkers for Diagnosis

The biomarker analysis module implemented in the MetaboanalystR package was used on the MS peak intensities table for all the samples for detecting relevant features for diagnostic classification. The random forest method, a classification ensemble algorithm, was used for classification and feature selection models. To construct ROC curves, balanced sub-sampling and Monte Carlo cross-validation (MCCV) with two thirds (2/3) of the samples for training were used to evaluate feature importance. The test subgroup (1/3 of samples) was used to build a classification model for top n (1 to 100) important features. The performance and confidence interval of each model were calculated, repeating the procedure multiple times. The RF model produces a reduced list of features ranked by value of importance. All the features obtained here were then used in the annotation stage.

#### 4.3.3. Putative Identification of Lipids and Metabolomics Pathway Analysis

In addition to the putative identification using Progenesis QI described above, the mummichog V2 algorithm [58] was used for MS peaks, without prior annotation. This method identifies lipids based on mass-to-charge ratios (*m/z*), *p*-values, fold change, retention time and mixed analytical mode (positive and negative ions), which were used to interrogate the KEGG library. Molecular weight tolerance at 5 ppm and a customized adduct list were used. Only lipidic matched compounds with registered LipidMaps entries were kept. A final manually curated list of identified lipids was obtained using Progenesis QI putative identification and the mummichog-identified lipid list. Using the identified compound list, metabolomics pathway analysis (MetPA) was used to identify biological pathway impact associated with the differences between study groups.

#### 4.3.4. Performance Evaluation of Diagnostic Biomarkers Used for Prognostic Prediction

To assess whether the lipids identified as diagnostic biomarkers could also be predictive for prognostic classification, these lipids were used to build a random forest predictive model for the prognosis. The most relevant lipid was further individually evaluated as a diagnostic and prognostic individual biomarker. For a more stringent evaluation as a possible biomarker, the predictive model tested a subgroup of unlabeled samples. A random forest model was then trained with the labeled subgroup of samples for a single compound, thus alleviating the training bias for which it was initially selected in the diagnostic classification. ANOVA two-way was used for final clustering and visualization of lipid relevant to both diagnosis and prognosis categories.

## Figures and Tables

**Table 1:** Baseline characteristics of the study population.

		Sepsis			SIRS		Sepsis vs. SIRS
	N	Mean	SD	N	Mean	SD	*p*-Value
Age	21	55.52	19.79	21	48.00	17.44	0.20
BMI	21	25.10	4.92	21	24.96	3.49	0.92
SAPSIII	21	56.95	17.17	21	53.05	14.72	0.43
Risk of death (%)	21	38.33	29.39	21	29.03	24.01	0.27
SOFA score	21	5.14	2.95	21	6.43	3.11	0.18
Comorbidities							
Systemic hypertension	7	0.33	-	1	0.05	-	0.05
Diabetes mellitus	5	0.24	-	0	0.00	-	0.05
Dyslipidemia	0	0.00	-	0	0.00	-	1.00
Coronary insufficiency	1	0.48	-	0	0.00	-	1.00
COPD	4	0.19	-	0	0.00	-	0.11
Neoplasm	3	0.14	-	0	0.00	-	0.23
Organ dysfunction							
by patient	21	2.05	-	21	2.05	-	0.82
AP < 90 mmHg	10	0.48	-	16	0.76	-	0.11
Lactate > 20 mg/dL	11	0.52	-	13	0.62	-	0.76
AKI	5	0.24	-	6	0.29	-	1.00
Total bilirubin > 2 mg/dL	3	0.14	-	1	0.05	-	0.61
INR > 1.6	8	0.38	-	1	0.05	-	0.02
Platelets < 150,000/mm^3^	1	0.05	-	4	19.05	-	0.34
PaO_2_/FiO_2_ ratio < 300	5	0.24	-	1	4.76	-	0.18
Site of infection							
Pneumonia	7	0.33	-	-	-	-	-
Abdominal	9	0.43	-	-	-	-	-
UTI	1	0.05	-	-	-	-	-
Others	4	0.19	-	-	-	-	-
Outcome (death)							
ICU length of stay	21	7.91	5.99	21	10.81	6.90	0.15
Total outcome	7	0.33	-	7	0.33	-	1.00

N: number of patients measured for each characteristic; Mean: average value for quantitative characteristics and proportion value for qualitative; SD: standard deviation; SIRS: systemic inflammatory response syndrome; BMI: body mass index; SAPSIII: Simplified Acute Physiology Score; SOFA: Sequential Organ Failure Assessment; COPD: chronic obstructive pulmonary disease; AP: arterial pressure; mmHg: millimeters of mercury; mg/dL: milligrams per deciliter; mm^3^: cubic millimeter; AKI: acute kidney injury; INR: international normalized ratio; FIO_2_: fraction of inspired oxygen; PaO_2_: partial pressure of oxygen; UTI: urinary tract infection. ICU: intensive care unit.

**Table 2:** Relevant ions selected by random forest (RF) models (positive and negative ion mode) to the diagnostic classification of plasma from sepsis and SIRS diagnosed patients.

Measured *m/z*	Ion Mode	Adducts	Lipid Assignment	Proposed Formula	Mass Error (ppm)	Abundance Sepsis	Abundance SIRS
129.0555	-	M-H_2_O-H [1−]	Mevalonic acid ^a^	C_6_H_12_O_4_	−1.69	1385.62 (722.52)	1277.46 (743.76)
132.0657	+	M+H [1+]	2-amino-4-oxopentanoic acid ^a^	C_5_H_9_NO_3_	1.51	991.28 (466.89)	1013.26 (580.62)
133.0854	+	M+H [1+]	6-hydroxyhexanoic acid ^a^	C_6_H_12_O_3_	−3.76	679.72 (866.08)	782.53 (914.21)
238.1169	+	M+H [1+]	S-aminomethyldihydrolipoamide ^a^	C_9_H_20_N_2_OS_2_	0.39	1114.93 (320.52)	1135.13 (536.29)
282.1251	-	M−2H [2−]	Leukotriene F4 ^b^	C_28_H_44_N_2_O_8_S	−2.54	1154.50 (1659.83)	1066.37 (662.07)
288.2181	+	M+H [1+]	L-octanoylcarnitine ^b^	C_15_H_29_NO_4_	4.16	1452.21 (1113.79)	659.59 (430.61)
293.2119	-	M−H_2_O-H [1−]	13-L-hydroperoxylinoleic acid ^b^	C_18_H_32_O_4_	−1.08	891.92 (644.89)	751.29 (539.68)
295.2277	-	M−H [1−]	13S-hydroxyoctadecadienoic acid ^b^	C_18_H_32_O_3_	−0.68	909.30 (645.14)	492.81 (355.81)
303.2333	-	M−H [1−]	Arachidonic acid ^b^	C_20_H_32_O_2_	0.99	817.75 (713.45)	1104.24 (653.40)
326.2670	+	M+H−H_2_O [+1]	N-palmitoyl serine ^a^	C_19_H_37_NO_4_	−5.64	2286.56 (4267.87)	3535.59 (5678.59)
327.2332	-	M−H [1−]	Docosahexaenoic acid ^a^	C_22_H_32_O_2_	0.61	812.39 (508.09)	780.96 (365.68)
331.2280	+	M+H [1+]	17-hydroxyprogesterone ^b^	C_21_H_30_O_3_	3.62	1142.74 (400.95)	1133.59 (530.18)
335.2218	+	M+H [1+]	PGE2 1,15-lactone ^a^	C_20_H_30_O_4_	0.30	709.67 (263.66)	561.39 (187.29)
353.2326	+	M+H [+1]	Prostaglandin E2 ^b^	C_20_H_32_O_5_	1.13	1405.19 (1305.15)	698.16 (637.36)
367.1578	-	M−H [1−]	Dehydroepiandrosterone sulfate ^b^	C_19_H_28_O_5_S	−1.90	614.23 (463.79)	2599.17 (1954.71)
397.2051	-	M−H_2_O-H [−1]	7-[(2,4,6-trihydroxy-2,5,5,8a-tetramethyl-decahydronaphthalen-1-yl)methoxy]-2H-chromen-2-one ^a^	C_24_H_32_O_6_	7.28	436.61 (267.23)	1948.12 (1895.83)
400.3438	+	M+H [1+]	L-palmitoylcarnitine ^b^	C_23_H_45_NO_4_	4.25	1275.23 (1117.99)	684.14 (547.16)
422.3260	+	M+H [1+]	Gamma-linolenyl carnitine ^a^	C_25_H_43_NO_4_	−1.18	994.63 (388.35)	832.62 (254.73)
424.3432	+	M+H [1+]	Linoleyl carnitine ^b^	C_25_H_45_NO_4_	2.59	1087.23 (1266.79)	598.35 (520.68)
426.3589	+	M+ACN+H [+1]	Tetrahydropersin ^a^	C_23_H_44_O_4_	2.89	1146.89 (1099.49)	568.64 (508.05)
464.3016	-	M-H [1−]	Glycocholic acid ^a^	C_26_H_43_NO_6_	−0.43	1506.15 (4407.32)	639.15 (1149.97)
477.2132	+	M+H [1+]	2-methoxyestrone 3-glucuronide ^a^	C_25_H_32_O_9_	2.72	1057.75 (267.34)	1088.04 (224.43)
510.3940	+	M+H [1+]	LysoPC (O-18:0) ^b^	C_8_H_20_NO_6_PR	4.31	932.19 (572.29)	776.95 (514.12)
557.4584	−	M−H [−1]	FAHFA 36:4 ^a^	C_36_H_62_O_4_	1.62	760.47 (665.67)	1528.53 (1306.92)
582.5110	−	M+FA−H [−1]	Cer (d16:1/18:0) ^a^	C_34_H_67_NO_3_	1.38	1931.34 (2267.96)	593.15 (508.32)
610.5423	−	M+FA−H [−1]	Cer (d36:1) ^a^	C_36_H_71_NO_3_	1.32	1497.42 (786.64)	707.04 (471.59)
753.5293	−	M+FA−H [−1]	PG (O−32:0) ^a^	C_38_H_77_O_9_P	0.87	1374.76 (687.85)	458.97 (455.32)
760.5590	−	M+FA−H [−1]	AS 1-5 ^a^	C_40_H_77_NO_9_	1.34	1273.41 (530.64)	489.87 (320.17)
762.5650	−	M−H [−1]	PS (O−35:0) ^a^	C_41_H_82_NO_9_P	−0.64	1541.92 (1105.94)	674.73 (559.37)
834.5294	−	M−H [1−]	PS (16:0/16:0) ^b^	C_38_H_74_NO_10_P	−0.41	1351.20 (525.54)	733.77 (538.39)
856.5141	−	M+Na−2H [−1]	PS (40:6) ^b^	C_46_H_78_NO_10_P	3.69	1575.69 (1472.24)	463.90 (483.53)
908.6356	−	M+Na−2H [−1]	PS (43:1) ^b^	C_49_H_94_NO_10_P	−0.63	1575.01 (1318.04)	1016.98 (1203.54)
932.6353	−	M+FA−H [−1]	PC (44:7) ^a^	C_52_H_90_NO_8_P	−3.75	1854.09 (1614.94)	621.01 (482.13)

*m/z*: mass-to-charge ratio; LysoPC: Lysophosphatidylcholine; PC: phosphatidylcholine; PG: phosphatidylglycerol; PGE2: prostaglandin E2; FAHFA: branched fatty acid esters of hydroxy fatty acids. Corrected abundance expressed as mean (standard deviation); a: Level 2 and b: Level 3 of annotation (see Methods).

## Supplementary Materials

The following are available online at https://www.mdpi.com/2218-1989/10/9/359/s1, Figure S1: PCA with quality controls, Figure S2: Negative mode ROC for diagnosis, Figure S3: Positive mode ROC for diagnosis, Figure S4: ROC for prognosis, Table S1: Ranked lipids for prognosis, Table S2: Pathway impact analysis, Table S3: Logistic linear model for all baseline characteristics, Spreadsheet_1: Sepsis_SIRS negative mode, Spreadsheet_2: Sepsis_SIRS positive mode.

## Abbreviations

The following abbreviations are used in this manuscript:

SIRS	systemic inflammatory response syndrome
SOFA	sequential organ failure assessment
qSOFA	quick SOFA
APACHE evaluation	acute physiology and chronic health
PCT	procalcitonin
SD	standard deviation
BMI	body mass index
CRP	C-reactive protein
SAPS	simplified acute physiology score
COPD	chronic obstructive pulmonary disease
AP	arterial pressure
AKI	acute kidney injury
mmHg	millimeters of mercury
mg	milligram
dL	deciliter
INR	international normalized ratio
mm3	cubic millimeter
PaO2	partial pressure of oxygen
FiO2	fraction of inspired oxygen
ICU	intensive care unit
UTI	urinary tract infection
USF	Universidade São Francisco
QC	quality control
PCA	principal component analysis
AUC	area under curve
ROC	receiver operating characteristic
RF	random forest
MSI	Metabolomics Standards Initiative
QC-RFSC correction	quality control random forest based signal
IQR	interquartile range
MCCV	Monte Carlo cross-validation
MS	mass spectrometry
HMDB	Human Metabolome Database
PC	phosphatidylcholine
PG	phosphatidylglycerol
ANOVA	analysis of variance
UPLC	ultra performance liquid chromatography
ACN	MS data-independent acquisition
MS^E^	n acetonitrile
EDTA	ethylenediamine tetraacetic acid
QTOF	quadrupole time-of-flight mass spectrometry
FAHFA	fatty acid esters of hydroxy fatty acids
TCA	tricarboxylic acid cycle
GSL	glycosphigolipids
LC-MS	liquid chromatography–mass spectrometry
