# Supplementary figures and images for: Potential Lipid Signatures for Diagnosis and Prognosis of Sepsis and Systemic Inflammatory Response Syndrome
Source: Metabolites. 2020 Sep 1;10(9):359. doi: 10.3390/metabo10090359 (PMC7570015; doi:10.3390/metabo10090359)

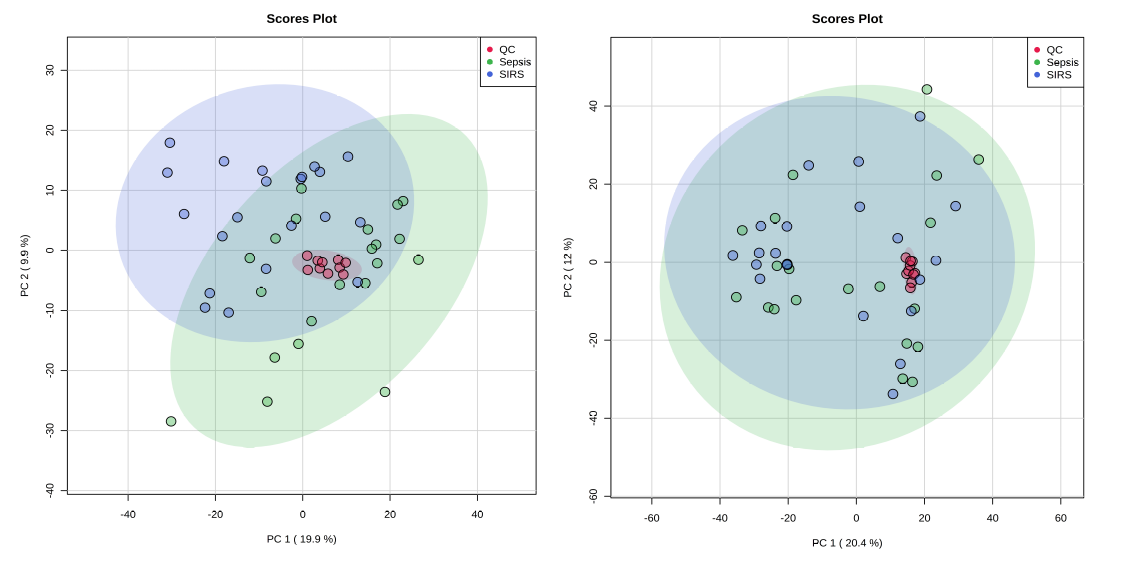

Supplement: Supplementary file 1 [file metabolites-10-00359-s001.zip › Figure S1 PCA with quality controls.png]

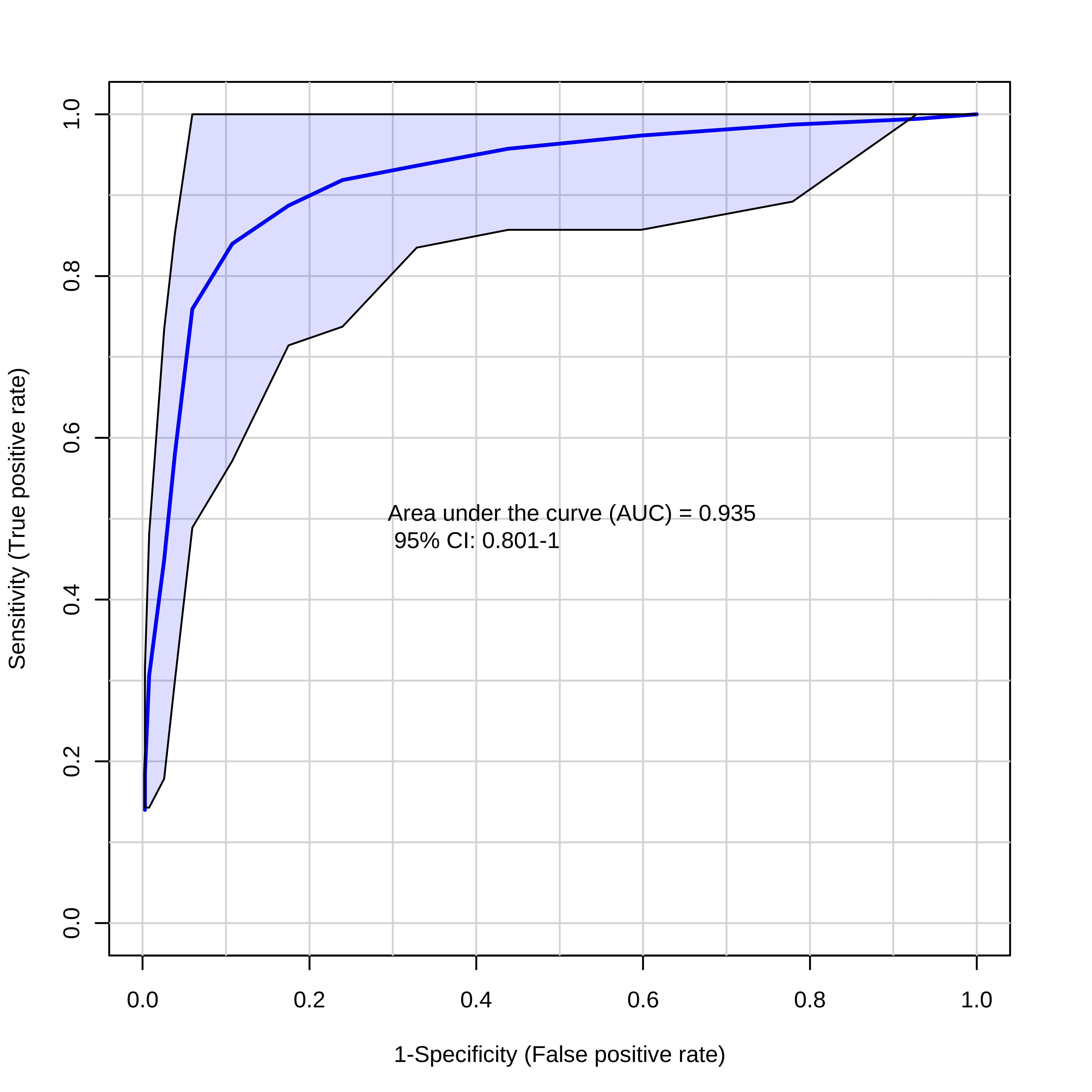

Supplement: Supplementary file 1 [file metabolites-10-00359-s001.zip › Figure S2 Negative mode ROC for diagnosis.png]

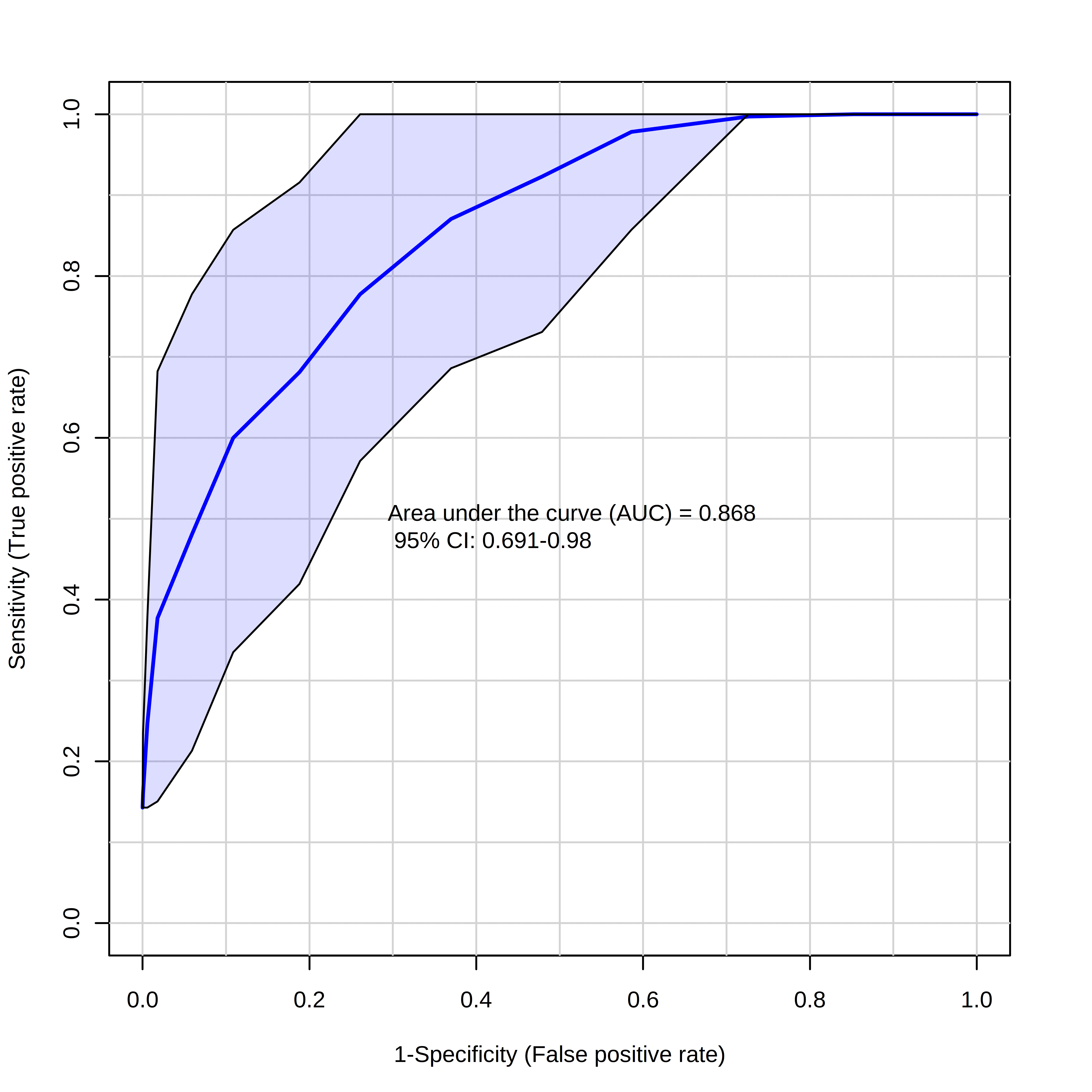

Supplement: Supplementary file 1 [file metabolites-10-00359-s001.zip › Figure S3 Positive mode ROC for diagnosis.png]

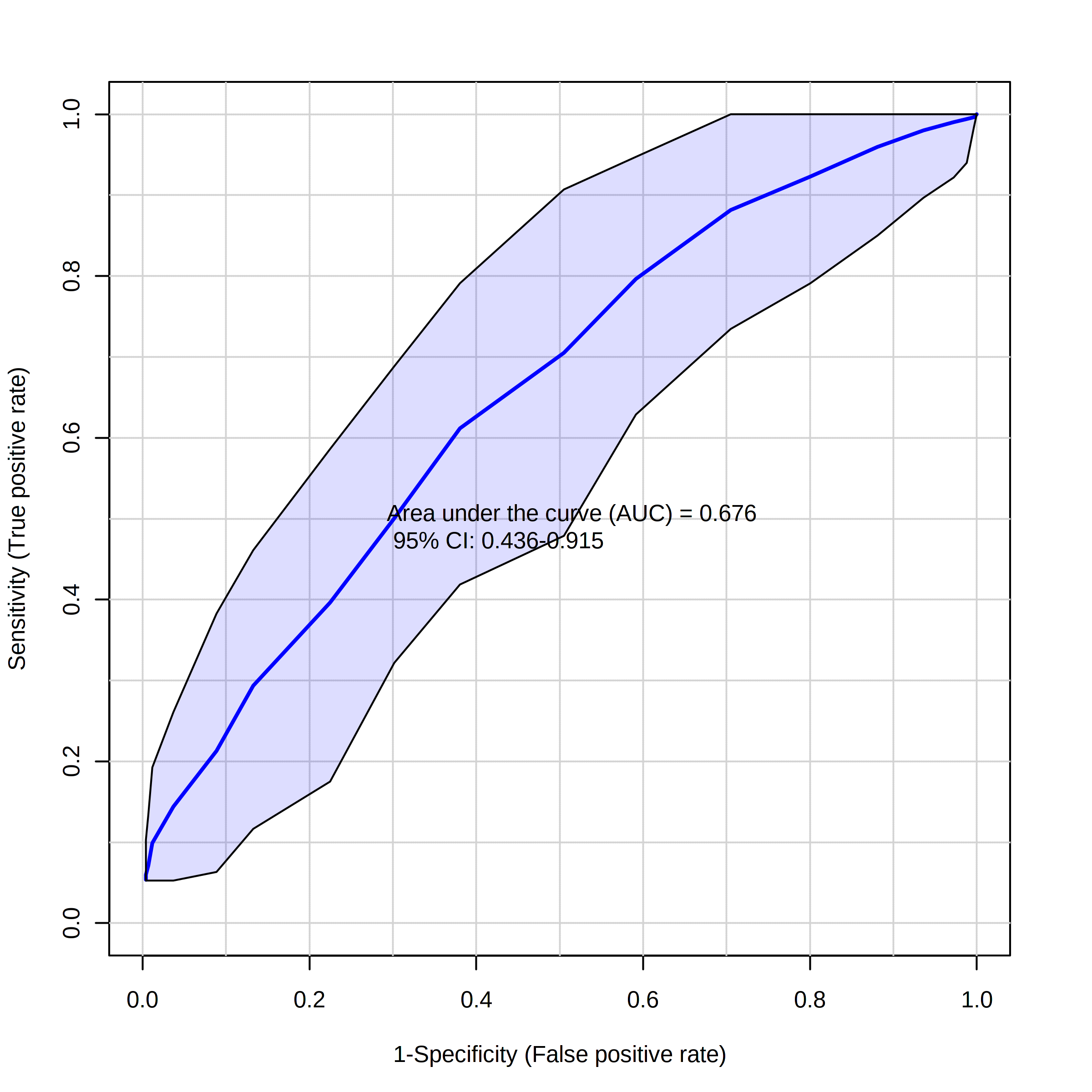

Supplement: Supplementary file 1 [file metabolites-10-00359-s001.zip › Figure S4 ROC for prognosis.png]
